# Knowledge, Attitudes, and Practices Regarding Probiotic Use in Enteral Feeding Among Intensive Care Unit Healthcare Professionals

**DOI:** 10.3390/healthcare14081033

**Published:** 2026-04-14

**Authors:** Khaled Tareg Hakami, Arwa S. Almasaudi, Areej Ali Alkhaldy, Batool Saad Almsaudi

**Affiliations:** 1 Department of Clinical Nutrition, Faculty of Applied Medical Sciences, King Abdulaziz University, Jeddah 21589, Saudi Arabia; khakami0036@stu.kau.edu.sa (K.T.H.);; 2 Department of Pharmacy, East Jeddah Hospital, Jeddah 22253, Saudi Arabia

**Keywords:** probiotics, enteral nutrition, intensive care unit, knowledge–attitudes–practices

## Abstract

**Background:** Probiotics have emerged as an effective therapeutic intervention in critically ill patients receiving enteral nutrition, yet their use remains inconsistent across intensive care units (ICUs). Understanding knowledge, attitudes, and practices (KAP) among healthcare professionals (HCPs) is essential for optimizing evidence-based probiotic administration in enteral nutrition, identifying perceived implementation barriers, and examining associations between KAP scores and study variables. **Methods:** A multicenter, cross-sectional online survey was administered to ICU physicians, nurses, clinical dietitians, pharmacists, and respiratory therapists. Participants completed a self-reported questionnaire assessing their knowledge of probiotic mechanisms, indications, and safety; attitudes toward probiotic therapy; and current practices in probiotic administration during enteral feeding. **Results:** A total of 935 ICU HCPs participated. Overall knowledge was insufficient, with only 33.2% achieving high knowledge scores (mean: 12.4/18 points), whereas attitudes were moderately favorable, with 35.5% demonstrating positive attitudes (mean: 23.9/30 points). A majority of respondents (58.7%) reported recommending or prescribing probiotics, most frequently clinical dietitians (84.5%). KAP varied significantly by profession, age group, and years of experience (*p* < 0.01). The most reported barriers were a lack of information about available probiotic products (73.2%), limited knowledge (41.2%), limited availability of clinically proven products (37.8%), and cost concerns (29.7%). **Conclusions:** Although ICU HCPs show interest and cautious acceptance of probiotics in enteral feeding, knowledge gaps, attitudinal variability, and practice inconsistencies persist across disciplines. These findings highlight the critical need for targeted, multidisciplinary educational interventions and the development of standardized, evidence-based institutional protocols to optimize probiotic use and improve patient outcomes.

## 1. Introduction

Patients admitted to the intensive care unit (ICU) frequently develop gastrointestinal complications for a variety of reasons, including broad-spectrum antibiotic exposure, proton pump inhibitor therapy, and changes in nutritional intake [1]. Together, these factors can lead to the disruption of gut microbiota known as gut dysbiosis, which is characterized by reduced microbial diversity, loss of beneficial bacteria, and overgrowth of potentially pathogenic organisms in the gastrointestinal tract. Gut dysbiosis has been associated with higher rates of nosocomial infection, multiple organ dysfunction syndrome, increased systemic inflammation, and overall poorer clinical outcomes [2].

Emerging strategies to address these problems include the use of probiotics, defined as live microorganisms which, when administered in adequate amounts, confer a health benefit on the host. Studies have demonstrated that probiotics can modulate epithelial and immune responses within ICU populations and mitigate gastrointestinal complications [3]. Furthermore, several systematic reviews and meta-analyses have suggested that probiotic administration in ICU patients may reduce the incidence of ventilator-associated pneumonia, decrease the duration of mechanical ventilation, and prevent ICU-acquired infections [4,5].

Although probiotics are generally safe to use, concerns regarding their safety in individuals with immunodeficiency or critical illness, particularly the risk of probiotic-associated bacteremia, have limited their use [6]. In addition, professional guidance emphasizes that probiotic effects are strain- and dose-specific, limiting our ability to extrapolate across products and indications [7].

Despite a growing body of evidence regarding probiotic use in critical care, studies and international surveys have demonstrated substantial variability in their adoption, dosing, and indications across ICUs, largely driven by inconsistent evidence and a lack of standardized guidelines [8,9]. A systematic review by Manzanares et al. revealed significant heterogeneity in probiotic formulations, dosing regimens, and patient selection criteria across randomized controlled trials in critical care settings [10].

This heterogeneity may result from differences among healthcare professionals (HCPs) with respect to knowledge of current evidence, attitudes toward the risks and benefits of probiotics, and the institutional protocols that regulate probiotic use. It is therefore essential to understand the knowledge, attitudes, and practices (KAP) of HCPs working in ICUs to identify evidence translation gaps, address misconceptions, and develop targeted educational interventions to maximize probiotic utilization during enteral feeding.

The beneficial effects of probiotics are mediated by multiple mechanisms, including competitive exclusion of pathogenic bacteria, enhancement of the intestinal epithelial barrier, modulation of host immune responses, and production of antimicrobial compounds such as bacteriocins and short-chain fatty acids. Together, these actions help restore gut microbiota balance, reduce intestinal permeability, and limit systemic inflammation in critically ill patients [11].

To date, no multicenter study has assessed KAP toward probiotic use among ICU HCPs in Saudi Arabia or the barriers to probiotic use within the country’s healthcare system. It is important to characterize KAP levels and perceived implementation barriers among ICU HCPs in Saudi Arabia to enhance patient care and guide future protocols pertaining to probiotic administration in ICUs. We therefore conducted a multicenter survey across several public hospitals in the Jeddah and Jazan Health Clusters to assess knowledge, attitudes, and self-reported practices regarding probiotic use in enteral feeding among ICU HCPs and identify perceived barriers to consistent probiotic implementation in the ICU setting.

## 2. Methods

### 2.1. Study Design

This study employed a multicenter, cross-sectional design to assess the KAP levels of ICU HCPs regarding the use of probiotics in adult patients receiving enteral nutrition. The survey was conducted in adult ICUs across two health cluster regions in Saudi Arabia (Jeddah and Jazan) and included 20 hospitals.

The data were collected over a two-month period between February and March 2025 using a questionnaire that was distributed and completed online by eligible ICU staff. Participants were required to answer all questions, resulting in no missing data.

### 2.2. Participants and Recruitment

All eligible ICU staff from Ministry of Health research units in Jeddah and Jazan, including physicians, nurses, clinical dietitians, pharmacists, and respiratory therapists directly involved in the care of adult ICU patients, were invited to participate in the study. The study included HCPs who were aged 20 years or older and had worked for at least three months in an adult ICU at the time of completing the survey. The study excluded staff in administrative-only roles and those on short ICU rotations with less than one month remaining at the time of invitation to ensure that responses reflected current ICU clinical practice.

### 2.3. Sample Size Calculation

An online sample size calculator (Raosoft, Raosoft Inc., Seattle, WA, USA) [12] was utilized to calculate the sample size using data obtained from the Saudi Ministry of Health (Statistical Yearbook, 2022) and based on an estimated total of approximately 2500 ICU-eligible HCPs in the Jeddah and Jazan health cluster regions [13]. The effective sample size was calculated to be 334, with a 95% confidence level, a 5% margin of error, and an assumed 50% response distribution.

### 2.4. Study Measures

The KAP survey used a structured, self-administered electronic questionnaire originally developed and validated by Ababneh et al. (2020) [14] to assess KAP regarding probiotics. The internal consistency of the questionnaire scales was evaluated using Cronbach’s alpha. The analysis yielded alpha coefficients of 0.71 for the Knowledge scale and 0.82 for the Attitude scale, indicating acceptable and good internal consistency, respectively.

The first section of the questionnaire collected sociodemographic and professional background information. This included age group, sex, professional category (physician, nurse, dietitian, pharmacist, or respiratory therapist), and years of ICU experience.

The second section assessed knowledge related to the use of probiotics in adult ICU patients receiving enteral nutrition. It contained nine items, which were scored 0 for an incorrect answer, 1 for an uncertain answer, and 2 for a correct answer. Next, the scores were classified into two categories, namely, low knowledge (below the third quartile: <75th percentile; score < 14) and high knowledge (above the third quartile: >75th percentile; score > 14). The maximum score for the knowledge section was 18.

The third section evaluated attitudes toward the use of probiotics in critically ill adult patients. This section included six statements rated on a five-point Likert scale, with response options of 1 (strongly disagree), 2 (disagree), 3 (neutral), 4 (agree), and 5 (strongly agree), such that higher scores indicated more favorable attitudes toward probiotic use. The total attitude score therefore ranged from 6 to 30. Next, the scores were classified into two categories, namely, negative attitudes (below the third quartile: <75th percentile; score < 26) and positive attitudes (above the third quartile: >75th percentile; score > 26).

The fourth section focused on practices and perceived barriers. Probiotic-related practice was assessed by a key question (yes/no) asking whether the respondent currently recommended or prescribed probiotics to ICU patients as part of their routine clinical care, which were scored 0 for a no answer, or 1 for a yes answer. Perceived barriers to probiotic use were evaluated using a list of 12 predefined obstacles, such as limited product availability, cost, lack of clear guidelines, safety concerns, doubts about effectiveness, and insufficient product information. Participants were allowed to select more than one barrier to reflect multiple coexisting challenges.

### 2.5. Data Collection

The survey of the study was disseminated to HCPs through the Ministry of Health research units in the Jeddah and Jazan Health Clusters. These units supplied study materials to hospital administrations, which notified the Public Relations and Training & Education departments to circulate the survey through official institutional channels.

These departments then informed the heads of the ICU and nursing departments, who in turn communicated the survey invitation to all eligible ICU staff (physicians, nurses, clinical dietitians, pharmacists, and respiratory therapists) and facilitated local dissemination via internal messaging systems and institutional email. Eligible staff received an authenticated electronic survey link via their institutional email accounts, with the electronic questionnaire serving as both the study instrument and distribution mode. Participation was voluntary. Completion and submission of the form were considered implied informed consent. To improve response, research units in each health cluster sent reminder messages through official channels at the beginning, one week after the initial invitation, and continuing until the end of the data collection period.

### 2.6. Statistical Analysis

Descriptive statistics were used to summarize the KAP survey data. All analyses were performed using IBM SPSS Statistics version 26. A two-sided significance level of α = 0.05 was adopted and exact *p* values were determined, with *p* < 0.001 reserved for very small values. Categorical variables, including demographic and professional characteristics, knowledge level (high vs. low), attitude level (positive vs. negative), practice (yes vs. no), and individual barriers, were summarized as frequencies and percentages. Knowledge and attitude scores were described using the mean ± standard deviation (SD) and the median with interquartile range (IQR). The internal consistency of the six-item attitude scale was assessed using Cronbach’s alpha (α).

The KAP scores were divided into two categories as per Bloom’s criteria as a cut-off of 75% of the score: low vs. high knowledge, positive vs. negative attitudes, and good vs. poor practices [15,16,17].

Probiotic-related practice was treated as a binary variable (yes/no) indicating whether the respondent reported recommending or prescribing probiotics to ICU patients. Perceived barriers to probiotic use were analyzed descriptively, with each of the 12 barrier items summarized as the number and percentage of respondents who endorsed that barrier. Pearson’s chi-squared test was used to examine bivariate associations between participant characteristics and KAP scores. Multivariable binary logistic regression was performed to identify factors independently associated with knowledge, attitude, and practice levels, with adjusted odds ratios (aORs), 95% confidence intervals, and *p* values reported. Spearman’s rank correlation was used to assess associations between knowledge, attitude, and practice scores.

### 2.7. Ethics

Ethical approval was obtained from the regional branches of the Ministry of Health for Jazan and Jeddah (approval No. 2469) and from the Research Ethics Committee of the King Abdulaziz University Hospital Biomedical Ethics Unit (ref. No. 36-25, NCBE registration No. HA-02-J-008).

## 3. Results

A total of 935 ICU HCPs participated in the survey. Table 1 summarizes the demographic and professional characteristics of the study participants. The majority of participants were women (*n* = 598, 64.0%), while men accounted for slightly over one-third of the total (*n* = 337, 36.0%). The predominant age group was 30–39 years old (*n* = 567, 60.6%). All core ICU disciplines were represented, with nursing staff comprising 25.7% of respondents (*n* = 240), physicians 22.7% (*n* = 212), clinical dietitians 20.6% (*n* = 193), pharmacists 17.6% (*n* = 165), and respiratory therapists 13.4% (*n* = 125). Overall, 360 participants (38.5%) had 5–10 years of professional experience and only 18 (1.9%) had more than 25 years of professional experience.

### 3.1. Knowledge of Probiotics in ICU Enteral Feeding

The participants demonstrated insufficient knowledge regarding probiotics in the context of enteral feeding. Using a predefined cut-off of ≥14/18 points on the total knowledge score (75th percentile of the observed distribution), 310 participants (33.2%) were classified as having high knowledge, while 625 (66.8%) were classified as having low knowledge (Table 2). The median total knowledge score was 12 (IQR = 3) and the mean was 12.39 (SD = 2.87), indicating that most respondents scored below the high-knowledge threshold. Item-level analysis revealed that safety-related aspects and clinical indications were better understood than basic concepts and mechanisms. A total of 563 respondents (60.2%) correctly identified that probiotics are associated with minimal risk for most patients. More than half of participants correctly answered the questions relating to allergies (*n* = 512, 54.8%), inflammatory bowel disease and irritable bowel syndrome (*n* = 492, 52.6%), urogenital conditions (*n* = 489, 52.3%), and strain-specific formulations (*n* = 471, 50.4%). Conversely, smaller percentages of respondents accurately recognized the fundamental definition of probiotics (*n* = 388, 41.5%), their prevalent delivery methods (*n* = 409, 43.7%), their capacity to influence immune responses (*n* = 442, 47.3%), and their involvement in diarrhea and lactose intolerance (*n* = 440, 47.1%).

### 3.2. Attitudes Toward Probiotic Use in ICU Enteral Feeding

The participants demonstrated moderately favorable but not uniformly positive attitudes toward probiotic use in enteral feeding. Using the predefined cut-off of ≥26/30 points on the total attitude score, 332 participants (35.5%) were classified as having a positive attitude, while 603 (64.5%) were classified as having a negative attitude (Table 3). The median total attitude score was 24 (IQR = 5) and the mean was 23.9 (SD = 4.10), indicating that most respondents scored below the positive-attitude threshold. Item-level analysis suggested very high interest in further education about probiotics. The vast majority of participants reported being interested in knowing more about probiotics (*n* = 833, 89.1%) and felt that they would benefit from education or workshops on probiotic use (*n* = 839, 89.7%). Relatively few respondents expressed strong endorsement of probiotics as a therapeutic modality. Only 228 participants (24.4%) concurred that probiotics significantly contributed to the treatment of various diseases, and 199 (21.3%) endorsed the utilization of live organisms in the management of medical conditions.

### 3.3. Probiotic-Related Practices Among ICU HCPs

Overall, 549 respondents (58.7%) reported that they recommended or prescribed probiotics to patients, while 386 (41.3%) reported that they did not (Table 4). Probiotic-related practices differed by profession. Among the physicians, 136 out of 212 (64.2%) reported recommending or prescribing probiotics. Among the nurses, 106 out of 240 (44.2%) reported doing so. Among the pharmacists, 111 out of 165 (67.3%) reported recommending or prescribing probiotics. Among the clinical dietitians, 163 out of 193 (84.5%) reported recommending or prescribing probiotics, and among the respiratory therapists, 33 out of 125 (26.4%) reported recommending or prescribing them. Among all respondents who reported using probiotics (*n* = 549), 163 (29.7%) were clinical dietitians, 136 (24.8%) were physicians, 111 (20.2%) were pharmacists, 106 (19.3%) were nurses, and 33 (6.0%) were respiratory therapists. The association between profession and probiotic-related practices was statistically significant (*p* < 0.001).

### 3.4. Perceived Barriers to Probiotic Use Among ICU HCPs

The participants reported multiple barriers to incorporating probiotics into routine practice (Table 5). The most frequently cited barrier was a lack of information about available probiotic products, which was reported by 684 participants (73.2%). In addition, 385 respondents (41.2%) indicated that they had little or no idea about probiotics, while 353 (37.8%) cited limited or no availability of clinically proven preparations. Concerns about safety, cost, and product quality were also common. A total of 305 respondents (32.6%) perceived no safety data, and 278 (29.7%) considered probiotics to be expensive.

### 3.5. Associations Between Demographic/Professional Characteristics and KAP Scores

The associations between the KAP scores and study variables are presented in Table 6. The analysis revealed that age, profession, and years of experience were each linked to at least one KAP domain. Age was significantly related to knowledge (*p* = 0.026) and attitudes (*p* < 0.001), but not to probiotic-related practices (*p* = 0.066). Sex was significantly linked only to attitudes (*p* < 0.001), with no significant links to knowledge (*p* = 0.537) or practices (*p* > 0.397). Profession displayed significant links with all three KAP domains (*p* < 0.001), with clinical dietitians exhibiting the highest rate of probiotic-related practices (84.5%) and respiratory therapists showing the lowest rate (26.4%). The number of years of experience was significantly linked to knowledge (*p* < 0.001) and attitudes (*p* < 0.001), but not practices (*p* = 0.178).

Multivariable logistic regression models were used to assess the potential determinants of KAP with respect to probiotic use in enteral feeding. All of the models included the age group, sex, profession, and years of experience as covariates. The reference categories were 20–29 years, male, physician, and <5 years of experience (Table 7).

In the knowledge model, a greater age was generally linked to higher odds of possessing a high level of knowledge. Participants aged 30–39 years and 40–49 years exhibited significantly greater odds of high knowledge compared with those aged 20–29 years (aOR = 1.844, 95% CI: 1.068–3.184, *p* = 0.028; and aOR = 2.861, 95% CI: 1.292–6.333, *p* = 0.010, respectively). Profession was also a key predictor: clinical dietitians displayed higher odds of high knowledge than physicians (aOR = 2.245, 95% CI: 1.456–3.463, *p* < 0.001). Sex and most years of experience levels were not significantly related to knowledge; however, participants with 21–25 years of experience showed notably lower odds of high knowledge (aOR = 0.227, 95% CI: 0.060–0.857, *p* = 0.029).

In the attitude model, years of experience emerged as the leading independent predictor of a positive attitude. Respondents with 5–10 and 11–15 years of experience displayed significantly greater odds of positive attitudes compared with those with <5 years of experience (aOR = 2.310, 95% CI: 1.190–4.490, *p* = 0.014; and aOR = 2.490, 95% CI: 1.160–5.340, *p* = 0.019, respectively). After adjustment, age, sex, and profession were not independently linked to attitude (all *p* > 0.05).

In the practice model, profession showed the strongest independent association with probiotic-related practices. Compared with physicians, nurses and respiratory therapists had significantly lower odds of reporting probiotic use (aOR = 0.384, 95% CI: 0.252–0.584, *p* < 0.001; and aOR = 0.185, 95% CI: 0.112–0.304, *p* < 0.001, respectively). In contrast, clinical dietitians exhibited much higher odds of probiotic-related practices (aOR = 2.898, 95% CI: 1.776–4.730, *p* < 0.001).

Years of experience also played a role independently: respondents with 5–10 and 11–15 years of experience had higher odds of probiotic-related practices compared with those possessing < 5 years of experience (aOR = 1.872, 95% CI: 1.119–3.132, *p* = 0.017; and aOR = 2.062, 95% CI: 1.145–3.714, *p* = 0.016, respectively), while the results for the groups with more years of experience were not statistically significant. After adjusting for profession and experience, age and sex were not independently linked to probiotic-related practices.

The correlation coefficients between KAP with respect to probiotic use in enteral feeding are presented in Table 8. The correlation between knowledge score and practice score was minimal but statistically significant (ρ = 0.065, *p* = 0.049), indicating a slight tendency for higher knowledge scores to be associated with reporting probiotic use. The relationship between attitude score and practice score was stronger, albeit still modest (ρ = 0.183, *p* < 0.001), indicating that more positive attitudes were more consistently associated with reporting probiotic use. Overall, these findings suggest a stronger connection between attitudes and practices than between knowledge and practices. The correlation between knowledge and attitude scores was not significant (ρ = 0.007, *p* = 0829), indicating no meaningful linear association between knowledge level and attitudinal favorability toward probiotic use.

## 4. Discussion

This multicenter study is the first to examine KAP levels with respect to probiotic use in enteral feeding among ICU HCPs in Saudi Arabia. Despite 58.7% (*n* = 549) of participants reporting probiotic use in practice, only 33.2% (*n* = 310) achieved high knowledge scores (≥14/18 points, mean 12.39 ± 2.87). Safety concerns and a lack of standardized protocols were found to impede evidence-based implementation. These findings have important implications for patient safety and rational probiotic use in critically ill populations, particularly given the strain- and dose-specific nature of probiotic efficacy emphasized in current guidelines [18]. Similarly, in a national cross-sectional survey, HCPs in Saudi Arabia demonstrated limited probiotic knowledge, with considerable variation by specialty, region, and profession [19]. The study found that only 14.3% of participants had “good” knowledge and less than 3% displayed “very good” knowledge.

Compared with Ababneh et al.’s study, our results differed as they reported that about half of the HCPs (51.5%) had fair knowledge, about 59% held positive attitudes, and 41% had ever recommended or prescribed probiotics, with the main barriers being lack of product information (90.5%) and limited familiarity (85.3%). In contrast, our study showed lower knowledge (only 33.2% achieved high knowledge) and fewer positive attitudes (35.5%), however, a larger proportion (58.7%) reported recommending or prescribing probiotics. These differences likely reflect differences in study population and setting. In addition, our study specifically surveyed ICU healthcare professionals, whereas Ababneh et al. sampled a broader HCP population across hospital departments. Moreover, with a large ICU sample (*n* = 935 vs. *n* = 205), our study may yield more precise, generalizable estimates for critical-care settings and allows for robust subgroup analyses. Notably, we documented the central role of clinical dietitians in recommending/prescribing probiotics within ICUs and quantified profession-specific prescribing patterns under ICU practice.

Furthermore, professional role was found to be an important determinant of knowledge. Clinical dietitians were more likely than other disciplines to achieve high knowledge scores, even after adjustment for age, sex, and experience, and pharmacists also tended to report stronger factual understanding than nurses and respiratory therapists. The lower odds of practice among nurses and respiratory therapists are likely attributable to their professional roles and scope of practice. Physicians, clinical dietitians, and pharmacists are typically responsible for prescribing and selecting therapeutic agents, including probiotics. In contrast, nurses and respiratory therapists are primarily involved in the administration and monitoring of prescribed treatments. Therefore, they have less autonomy in initiating probiotic use, which is reflected in their lower reported practice rates. This pattern aligns with findings from Saudi Arabia that indicate that nutrition and pharmacy professionals possess greater probiotic knowledge than other healthcare workers [19,20,21].

International studies similarly suggest that greater exposure to nutrition-focused training and product-related information is associated with a better understanding of probiotics and more frequent clinical use [22]. It is plausible that the dietitians and pharmacists surveyed in the present study were more frequently exposed to probiotic-related evidence, guidelines, and product information during formal education and routine practice, whereas other ICU staff encountered probiotics more sporadically.

Age and experience also appeared to influence knowledge. Mid-career professionals (30–39 and 40–49 years old), who typically had more ICU exposure, were more likely to be classified as having high knowledge than younger colleagues, suggesting that repeated contact with enteral nutrition and adjunctive therapies may gradually increase familiarity with probiotics. However, the presence of substantial numbers of low-knowledge respondents in all age and experience categories, together with the small effect sizes for these associations, indicates that experience alone is not sufficient to ensure up-to-date understanding. Without structured education and clear institutional guidance, HCPs may continue to rely on fragmented or outdated information.

The multivariable logistic regression identified an interesting finding whereby ICU HCPs with 21–25 years of experience had significantly lower odds of high knowledge compared with those with fewer than five years of experience (aOR = 0.227, 95% CI: 0.060–0.857, *p* = 0.029). This may be because senior professionals may have completed their formal training before probiotics became a prominent topic and may be less exposed to recent evidence-based practice updates compared to mid-career colleagues. However, this finding warrants cautious interpretation, as this subgroup comprised only 26 participants.

Furthermore, ICU HCPs were interested in and cautious about administering probiotics to critically ill adults. Only about one-third of respondents reached the predefined threshold for a positive overall attitude score, yet the vast majority reported that they were interested in learning more about probiotics and believed that they would benefit from further education or workshops. This pattern suggests that probiotics are viewed as a potentially useful, albeit not yet fully integrated, component of ICU care, particularly in the high-risk context of critical illness.

There is agreement with these findings in global surveys. One study found that 79% of health professionals advised patients to take probiotics, and 57.5% were interested in learning more [23]. In Türkiye, most physicians and pharmacists were found to possess favorable attitudes; 70.7% of pharmacists and 38.2% of physicians reported no objection to recommending probiotics [24]. In the United Arab Emirates, pharmacists generally held positive views and almost all considered probiotics safe, particularly in the context of gastrointestinal health [25].

The lack of a significant correlation between knowledge and attitude (ρ = 0.007, *p* = 0.829) suggests that HCPs’ attitudes toward probiotics may be shaped more by clinical experience, institutional culture, or interest in novel therapies rather than by their formal knowledge.

Multivariable analysis revealed that intermediate ICU experience (5–15 years) predicted positive attitudes toward probiotics, independent of age, sex, and profession. Mid-career HCPs may have sufficient exposure to enteral nutrition therapies to recognize the potential benefits of probiotics while remaining engaged in evidence-based practice updates. Junior staff may defer to established routines due to uncertainty, while senior HCPs may adhere to long-standing care patterns. Unlike knowledge and practices, which showed clear professional gradients, attitudes appear shaped more by individual experience and local ICU culture than by discipline.

Although 58.7% of ICU professionals reported recommending probiotics, significant disciplinary differences were observed. Clinical dietitians, pharmacists, and physicians were the most frequent users, while nurses and respiratory therapists reported substantially lower usage, a difference that persisted even after adjusting for age, sex, and experience. This pattern reflects professional roles: dietitians and physicians typically lead enteral nutrition decisions, pharmacists manage product selection, and nurses and respiratory therapists primarily handle administration and monitoring rather than prescription. Consequently, probiotics are perceived as primarily within the domain of nutrition, pharmacy, and medical personnel, with other professions interacting mainly in implementation roles.

Similar profession-related gradients have been reported in regional studies, where dietitians, gastroenterologists, and pharmacists tend to use probiotics more frequently than other HCPs and often describe their approach as cautious and selective [19,21,26]. International surveys likewise show that probiotics are used inconsistently across disciplines and indications, frequently in the absence of standardized protocols [14,23,25].

Several modifiable barriers were identified. The most frequently reported obstacle was a perceived lack of accessible information about probiotic products, including available brands, strains, and their evidence base. Many respondents reported only a vague understanding of probiotics or felt that clinically proven preparations were limited or unavailable in their institutions. Some earlier KAP work from Saudi Arabia revealed that the most common obstacle is insufficient education and training, particularly in certain regions and specialties [19,21]. The lack of standardized educational materials and clinical protocols adds to this, leading to inconsistent practice patterns [20].

Concerns regarding safety, product quality, and regulation were also common. Around one-third of respondents expressed unease about administering live organisms to critically ill patients, while others highlighted doubts about quality or noted that dietary supplements are not regulated to the same standard as conventional medicines. Some regarded the clinical use of probiotics as controversial or were worried about infection risk. Similar patterns of skepticism regarding the robustness of the evidence base, variability in product quality, and uncertainty about the risk–benefit balance, especially in high-acuity or immunocompromised populations, have been reported in regional and international studies [20,27,28]. Cost-related and practical challenges posed additional barriers to the utilization of probiotics. Nearly one-third of participants perceived probiotics as expensive, some believed traditional yogurt to be as effective as commercial probiotic products, and others felt that standard therapies offered similar benefits. Previous surveys in Saudi Arabia and elsewhere have similarly identified perceived cost, lack of authority to prescribe, logistical constraints, and reliance on familiar treatments as important barriers to probiotic use [21,23,25,29].

### 4.1. Clinical and Policy Implications

Our findings reveal that overcoming the barriers to appropriate probiotic use in the ICU requires a multi-faceted strategy that extends beyond individual education. The practice variability observed is largely a systemic issue, pointing to several key clinical and policy recommendations.

First, a strong framework of institutional policies is the cornerstone of effective practice. Hospitals should establish an approved formulary of evidence-based probiotic products, prioritizing those with third-party quality certification to address concerns about product consistency and safety. This formulary must be supported by clear, standardized institutional protocols that guide clinicians on appropriate indications, dosing, administration, and patient selection criteria.

Second, to directly address safety concerns and build trust, implementing a system to monitor for any adverse events associated with probiotic use ensures patient safety and generates crucial local outcome data. Sharing these data can validate efficacy within the institution and foster greater confidence among frontline staff.

Finally, these policy-level interventions empower targeted education. By aligning educational initiatives with the institution’s specific formulary and protocols, training becomes more practical and relevant, effectively bridging the gap between abstract knowledge and daily clinical practice. Together, these strategies can systematically transform cautious, heterogeneous use into a confident, standardized, and evidence-based approach.

### 4.2. Strengths and Limitations

This study has several strengths and limitations. The survey included a large, diverse sample of ICU HCPs from multiple centers, enhancing the representativeness of the resulting findings across adult ICUs in Saudi Arabia. However, several limitations should be considered. First, the cross-sectional design precludes causal inference between knowledge, attitudes, and practices regarding probiotic use. Second, self-reported data may introduce recall bias and social desirability bias, potentially leading to the overestimation of knowledge and more favorable presentation of practices than actual clinical behavior. Third, although the study included multiple centers and disciplines, the findings may not fully generalize to pediatric ICUs, hospitals with different resource levels, or healthcare systems outside of Saudi Arabia with different training structures and scopes of practice. Fourth, although our sample included a relatively even distribution of professional groups, this may not reflect the actual staffing mix in many ICUs and could therefore influence profession-specific prevalence comparisons. Therefore, we report profession-specific sample sizes and percentages in Section 3. Thus, readers should interpret profession-level prevalence estimates with caution when generalizing to ICUs that have different workforce structures. Finally, the knowledge assessment instrument; for example, one knowledge item that assessed the general safety of probiotic use (e.g., “probiotics are associated with minimal risks for most patients”) was designed as a broad foundational KAP tool applicable across all ICU disciplines and may not have fully captured advanced, strain-specific risks, infection risk in immunocompromised patients, and device/enteral feeding considerations. Therefore, readers should interpret the knowledge findings with caution. Future studies employing discipline-specific or tiered knowledge assessments incorporating questions on CFU dosing, intervention duration, and ICU-specific strain selection would provide more granular insights into clinical competence.

## 5. Conclusions

Overall, our results indicate cautious openness toward probiotic use among ICU HCPs, with high interest in further education. However, this enthusiasm contrasts with knowledge gaps regarding probiotic mechanisms, strain-specific effects, evidence-based indications, and safety considerations in critically ill patients. Notable disparities emerged across professional groups, with clinical dietitians demonstrating superior knowledge compared with other specialties, likely reflecting their direct involvement in enteral feeding management. These findings highlight the need for targeted, multidisciplinary educational interventions and the development of standardized, evidence-based institutional protocols to bridge the knowledge–practice gap.

## Figures and Tables

**Table 1:** Demographic and professional characteristics of the participants (*n* = 935).

Characteristic	Category	*n* (%)
Gender	Male	337 (36.0)
	Female	598 (64.0)
Age	20–29 years	240 (25.7)
	30–39 years	567 (60.6)
	40–49 years	109 (11.7)
	≥50 years	19 (2.0)
Profession	Physician	212 (22.7)
	Nurse	240 (25.7)
	Pharmacist	165 (17.6)
	Clinical dietitian	193 (20.6)
	Respiratory therapist	125 (13.4)
Years of experience	<5 years	213 (22.8)
	5–10 years	360 (38.5)
	11–15 years	237 (25.3)
	16–20 years	81 (8.7)
	21–25 years	26 (2.8)
	>25 years	18 (1.9)

**Table 2:** Responses to questions assessing the knowledge of participants about probiotic use in ICU enteral feeding (*n* = 935).

Question	Possible Range *	Low Knowledge *n* (%)	High Knowledge *n* (%)
Probiotics are live microorganisms providing a health benefit when taken in adequate amounts ^a^	0–2	547 (58.5)	388 (41.5)
Probiotics are consumed as supplements or probiotic-fortified foods ^a^	0–2	526 (56.3)	409 (43.7)
Probiotics cannot modulate immune responses ^b^	0–2	493 (52.7)	442 (47.3)
Some probiotic products have clinically proven beneficial effects in diarrhea and lactose intolerance ^a^	0–2	495 (52.9)	440 (47.1)
Some probiotic products are effective in inflammatory bowel disease and irritable bowel syndrome ^a^	0–2	443 (47.4)	492 (52.6)
Probiotics cannot play a role in urogenital conditions ^b^	0–2	446 (47.7)	489 (52.3)
Probiotics cannot be effective in treating allergies ^b^	0–2	423 (45.2)	512 (54.8)
Probiotics are available in different forms and strains; each one has different effects ^a^	0–2	464 (49.6)	471 (50.4)
There are minimal risks associated with the clinical use of probiotics for most patients ^a^	0–2	372 (39.8)	563 (60.2)
Total knowledge questions	0–18	625 (66.8)	310 (33.2)
Knowledge score ^†^			
Minimum score = 0			
Maximum score = 18			
Median (IQR) = 12 (3)			
Mean (SD) = 12.39 (2.87)			

^a^ Response should be “correct” (the statement is true; agreement was coded as the correct answer). ^b^ Response should be “incorrect” (the statement is false; disagreement was coded as the correct answer). * Each question was scored 0 for an incorrect answer, 1 for an uncertain answer, and 2 for a correct answer, giving a possible range of 0–2 per question. ^†^ For item-level analysis, low knowledge includes responses scored 0 or 1, and high knowledge includes responses scored 2. The total knowledge score (0–18) is the sum of all nine items. Low overall knowledge was defined as a total score of <14 (below the 75th percentile), and high overall knowledge was defined as a total score of ≥14 (at or above the 75th percentile).

**Table 3:** Responses to questions assessing the attitudes of participants toward probiotic use in ICU enteral feeding (*n* = 935).

Statement	Possible Range *	Negative Attitude *n* (%)	Positive Attitude *n* (%)
I am interested in knowing more about probiotics	1–5	102 (10.9)	833 (89.1)
I would benefit from education or workshops related to the use of probiotics	1–5	96 (10.3)	839 (89.7)
I acknowledge that probiotics play a significant role in treating various diseases	1–5	707 (75.6)	228 (24.4)
I accept using live organisms in the management of medical conditions	1–5	736 (78.7)	199 (21.3)
I recommend probiotics without any concerns	1–5	533 (57.0)	402 (43.0)
I prefer prescribing more probiotics than antibiotics in conditions that could be treated by antibiotics and probiotics	1–5	517 (55.3)	418 (44.7)
Total attitude statements	6–30	603 (64.5)	332 (35.5)
Attitude score			
Minimum score = 6			
Maximum score = 30			
Median (IQR) = 24 (5)			
Mean (SD) = 23.9 (4.10)			

* The possible range of 1–5 represents responses from 1 for “strongly disagree” to 5 for “strongly agree” for the positive attitude statements. Negative attitudes were defined as scores below the third quartile (below the 75th percentile; score < 26), whereas positive attitudes were defined as scores at or above the third quartile (at or above the 75th percentile; score ≥ 26). The highest possible attitude score was 30.

**Table 4:** Probiotic-related practices by profession among participants (*n* = 935).

Profession	Recommending or Prescribing Probiotics to Patients		Total
	Yes *	No	
Physician	136 (64.2)	76 (35.8)	212 (22.7)
Nurse	106 (44.2)	134 (55.8)	240 (25.7)
Pharmacist	111 (67.3)	54 (32.7)	165 (17.6)
Clinical dietitian	163 (84.5)	30 (15.5)	193 (20.6)
Respiratory therapist	33 (26.4)	92 (73.6)	125 (13.4)
Total	549 (58.7)	386 (41.3)	935 (100)

* The percentages listed in the rows were calculated for each profession.

**Table 5:** Perceived barriers to probiotic use among participants (*n* = 935).

Barrier	*n* (%)
Lack of information regarding available probiotic products	684 (73.2)
Little or no idea about probiotics	385 (41.2)
Limited or no availability of clinically proven probiotic products	353 (37.8)
Clinical use of probiotics is controversial	220 (23.5)
Doubts in the quality of probiotic products	173 (18.5)
No data on the safety of probiotics	305 (32.6)
Dietary supplements, like probiotics, are not regulated by the FDA	109 (11.7)
Probiotics are expensive	278 (29.7)
Traditional yogurts are as effective as probiotic products	123 (13.2)
The efficacy of probiotics is inferior to or does not provide additional benefit over standard therapeutics	113 (12.1)
Negative experience with prior use of probiotics	84 (9.0)
Risk of infection due to probiotic use	108 (11.6)

**Table 6:** Associations between demographics, professional characteristics, years of experience, and KAP scores with respect to probiotic use in enteral feeding.

Variable	Knowledge Score			Attitude Score			Practice Score		
	Low	High	*p* Value	Negative	Positive	*p* Value	Yes	No	*p* Value
	*n* (%)	*n* (%)		*n* (%)	*n* (%)		*n* (%)	*n* (%)	
Age (years)									
20–29	179 (74.6)	61 (25.4)	**0.026**	178 (74.2)	62 (25.8)	<0.001	124 (51.7)	116 (48.3)	**0.066**
30–39	367 (64.7)	200 (35.3)		361 (63.7)	206 (36.3)		343 (60.5)	224 (39.5)	
40–49	68 (62.4)	41 (37.6)		52 (47.7)	57 (52.3)		70 (64.2)	39 (35.8)	
≥50	11 (57.9)	8 (42.1)		12 (63.2)	7 (36.8)		12 (63.2)	7 (36.8)	
Sex									
Male	221 (65.6)	116 (34.4)	0.537	247 (73.3)	90 (26.7)	**<0.001**	204 (60.5)	133 (39.5)	0.397
Female	404 (67.6)	194 (32.4)		356 (59.5)	242 (40.5)		345 (57.7)	253 (42.3)	
Professions									
Physician	149 (70.3)	63 (29.7)	**<0.001**	119 (56.1)	93 (43.9)	**<0.001**	136 (64.2)	76 (35.8)	**<0.001**
Nurse	182 (75.8)	58 (24.2)		142 (59.2)	98 (40.8)		106 (44.2)	134 (55.8)	
Pharmacist	105 (63.6)	60 (36.4)		126 (76.4)	39 (23.6)		111 (67.3)	54 (32.7)	
Clinical dietitian	102 (52.9)	91 (47.1)		110 (57)	83 (43)		163 (84.5)	30 (15.5)	
Respiratory therapist	95 (76)	30 (24)		103 (82.4)	22 (17.6)		33 (26.4)	92 (73.6)	
Experience									
<5 years	174 (81.7)	39 (18.3)	**<0.001**	177 (83.1)	36 (16.9)	**<0.001**	109 (51.2)	104 (48.8)	0.178
5–10 years	248 (68.9)	112 (31.1)		234 (65.0)	126 (35.0)		218 (60.6)	142 (39.4)	
11–15 years	151 (63.7)	86 (36.3)		132 (55.7)	105 (44.3)		143 (60.3)	94 (39.7)	
16–20 years	34 (42.0)	47 (58.0)		31 (38.3)	50 (61.7)		51 (63.0)	30 (37.0)	
21–25 years	10 (38.5)	16 (61.5)		15 (57.7)	11 (42.3)		18 (69.2)	8 (30.8)	
>25 years	8 (44.4)	10 (55.6)		14 (77.8)	4 (22.2)		10 (55.6)	8 (44.4)	

*p* values of <0.05 are highlighted in bold.

**Table 7:** Multivariable logistic regression for high knowledge, positive attitude, and probiotic-related practices (yes = 1).

Variable	Knowledge Score		Attitude Score		Practice Score	
	aOR (95% CI)	*p* Value	aOR (95% CI)	*p* Value	aOR (95% CI)	*p* Value
Age (years)						
20–29	1		1		1	
30–39	1.844 (1.068–3.184)	0.028	0.830 (0.430–1.590)	0.570	0.821 (0.504–1.338)	0.429
40–49	2.861 (1.292–6.333)	**0.010**	0.580 (0.220–1.560)	0.280	0.932 (0.431–2.014)	0.858
≥50	3.893 (0.702–21.596)	0.120	0.470 (0.070–3.150)	0.440	2.957 (0.468–18.673)	0.249
Sex						
Male	1		1		1	
Female	1.021 (0.742–1.405)	0.898	0.690 (0.450–1.060)	0.09	1.037 (0.753–1.428)	0.823
Professions						
Physician	1		1		1	
Nurse	0.952 (0.599–1.513)	0.834	0.840 (0.480–1.490)	0.560	0.384 (0.252–0.584)	**<0.001**
Pharmacist	1.325 (0.832–2.110)	0.235	0.810 (0.450–1.470)	0.480	1.079 (0.694–1.679)	0.736
Clinical dietitian	2.245 (1.456–3.463)	**<0.001**	1.050 (0.580–1.910)	0.870	2.898 (1.776–4.730)	**<0.001**
Respiratory therapist	0.942 (0.554–1.605)	0.827	0.740 (0.390–1.400)	0.360	0.185 (0.112–0.304)	**<0.001**
Experience						
<5 years	1		1		1	
5–10 years	0.833 (0.475–1.460)	0.523	2.310 (1.190–4.490)	0.014	1.872 (1.119–3.132)	**0.017**
11–15 years	0.708 (0.380–1.318)	0.276	2.490 (1.160–5.340)	0.019	2.062 (1.145–3.714)	**0.016**
16–20 years	0.636 (0.276–1.465)	0.288	1.950 (0.700–5.390)	0.200	1.815 (0.811–4.063)	0.147
21–25 years	0.227 (0.060–0.857)	**0.029**	2.340 (0.560–9.830)	0.250	3.146 (0.970–10.201)	0.056
>25 years	0.477 (0.146–1.557)	0.219	1.330 (0.200–8.910)	0.770	0.399 (0.065–2.468)	0.323

*p* values of <0.05 are highlighted in bold.

**Table 8:** Correlation coefficients between knowledge, attitudes, and practices with respect to probiotic use in enteral feeding.

Domain	Knowledge	Attitude	Practice
Knowledge	1		
Attitude	0.007 NS	1	
Practice	0.065 *	0.183 **	1

Spearman’s rank correlation coefficient. * Correlation was significant at the 0.05 level (*p* = 0.049). ** Correlation was significant at the 0.001 level (*p* < 0.001). NS = Not Significant (*p* = 0.829).

## Data Availability

The data presented in this study are available on request from the corresponding author.
